# Using heterogeneous sources of data and interpretability of prediction models to explain the characteristics of careless respondents in survey data

**DOI:** 10.1038/s41598-023-40209-2

**Published:** 2023-08-17

**Authors:** Leon Kopitar, Gregor Stiglic

**Affiliations:** 1 Faculty of Health Sciences, University of Maribor, Maribor, Slovenia; 2 Faculty of Electrical Engineering and Computer Science, University of Maribor, Maribor, Slovenia; 3 Usher Institute, University of Edinburgh, Edinburgh, UK

**Keywords:** Computational science, Scientific data, Computer science

## Abstract

Prior to further processing, completed questionnaires must be screened for the presence of careless respondents. Different people will respond to surveys in different ways. Some take the easy path and fill out the survey carelessly. The proportion of careless respondents determines the survey’s quality. As a result, identifying careless respondents is critical for the quality of obtained results. This study aims to explore the characteristics of careless respondents in survey data and evaluate the predictive power and interpretability of different types of data and indices of careless responding. The research question focuses on understanding the behavior of careless respondents and determining the effectiveness of various data sources in predicting their responses. Data from a three-month web-based survey on participants’ personality traits such as honesty-humility, emotionality, extraversion, agreeableness, conscientiousness and openness to experience was used in this study. Data for this study was taken from Schroeders et al.. The gradient boosting machine-based prediction model uses data from the answers, time spent for answering, demographic information on the respondents as well as some indices of careless responding from all three types of data. Prediction models were evaluated with tenfold cross-validation repeated a hundred times. Prediction models were compared based on balanced accuracy. Models’ explanations were provided with Shapley values. Compared with existing work, data fusion from multiple types of information had no noticeable effect on the performance of the gradient boosting machine model. Variables such as “I would never take a bribe, even if it was a lot”, average longstring, and total intra-individual response variability were found to be useful in distinguishing careless respondents. However, variables like “I would be tempted to use counterfeit money if I could get away with it” and intra-individual response variability of the first section of a survey showed limited effectiveness. Additionally, this study indicated that, whereas the psychometric synonym score has an immediate effect and is designed with the goal of identifying careless respondents when combined with other variables, it is not necessarily the optimal choice for fitting a gradient boosting machine model.

## Introduction

In the modern era, due to the hectic lifestyle of most people, surveys provide an easy and rapid way of collecting data that serves as feedback without any need for direct human-to-human interaction. Although conducting a survey is a simple solution to data collection in many cases, it also brings some drawbacks. Respondents with different backgrounds and mentalities will respond to surveys differently. Some recognize the importance of completing the survey and addressing each item, others simply take an easy road and complete it in a careless manner. Careless responding is categorized into several types: random respondents that respond at random choice, midpoint respondents who hold the higher probabilities for selecting middle categories, and fixed pattern respondents (e.g. 1, 2, 1, 2...)^1,2^.

The proportion of so-called careless respondents determines the quality of collected survey data. As reported by Credé, careless responding rates as low as 5% have the potential to significantly affect observed correlations compared to important study artifacts such as range restriction, dichotomization of continuous variables, and score unreliability^3^. As a result, the identification of careless respondents is essential for further improvements in this field of research.

Previous studies proposed various methods to detect careless respondents. In 1976, according to a study by Johnson, Jackson recommended an index, now known as the even-odd consistency score^4^. The even-odd consistency examines the relationship between scores obtained from the odd and even sections within different subscales^4^. Maniaci and Rogge reported that a cutoff value of less than 0.3 indicates careless responding^5^. Johnson also stated that Goldberg suggested a method based on psychometric antonyms , which are item pairs that are correlated highly negatively^4^. Based on that method, Meade and Craig developed the psychometric synonyms index , which on the other hand, are item pairs that are correlated highly positively^1^. Maniaci and Rogge observed that the most substantial average increase in power was achieved when the threshold values for indices of careless responding, specifically psychometric antonyms and psychometric synonyms, were below $-0.65$ and $-0.03$, respectively^5^. In the same study, they reported that the suggested cutoff value for psychometric antonyms ($<{-0.03}$^4,6^) resulted in 4–9% drop in power. According to the study of Nielsen et al., the even-odd consistency score and psychometric indexes are of limited usefulness when it comes to questionnaires with a small number of items and scales (e.g. thirty subscales)^7^.

Another index, the longstring index, was introduced in the study conducted by Johnson^4^. It is an extended sequence of continuous and identical answers provided by an individual. While Maniaci and Rogge reported that the longstring index of more than 7 is a great cutoff value for detecting careless responding^5^, Johnson points out that the longstring index is sensitive to responses that exhibit extreme consistency and cutoff value is difficult to determine^4^. Costa and McCrae offered recommendations for the maximum length of longstrings using the NEO-PI-R as a basis^8^. Another indices of careless responding, Mahalanobis distance has been demonstrated to be efficient at detecting careless respondents^9^. It assumes that responses significantly deviating from the sample norm (resulting in larger Mahalanobis distance) may indicate careless responding. The drawback is that it demonstrates efficiency solely when generating genuinely random responses^1^. In 2018, Dunn et al.^10^ introduced an indicator named intra-individual response variability and other more advanced methods, such as the systematic approach in a method named floodlight detection for careless respondents^11^. The drawbacks of intra-individual response variability include the necessity to calculate it across multiple constructs and reversely coded items. Furthermore, the presence of both low and high variability could potentially indicate careless responding^2,10,12^. Goldammer et al.^13^ found response time per item, personal reliability, psychometric synonyms/antonyms and Mahalanobis distance to be effective methods for detecting carelessness, on the contrary, longstring and intra-individual response variability were not significantly related to detection of careless respondents. In 2022, Wind and Wang^14^ conducted a study using Mokken scale analysis to detect carelessness in the survey, where they showed the robustness of Mokken scale analysis indicators of item quality to the presence of careless respondents. It is unknown how Mokken scale analysis performs on data with missing responses and to carelessness patterns other than random responses and overly consistent responding^14^. Arias et al. employed a factor mixture model specifically created to identify discrepancies in the way individuals respond to items that have varying semantic polarity^15^. Another study put forth a model based on item response theory that aims to identify and model careless responding at a detailed level, considering both the respondent and the specific item. Their model has the capability to detect various patterns of careless responding and contributes to a more comprehensive understanding of the item characteristics associated with its occurrence^16^. Ulitzsch et al. introduced a model-based approach that utilizes response time data from computer-administered questionnaires to simultaneously detect different manifestations of careless responding^17^. Their approach considers the characteristics of attentive response behavior on questionnaires by incorporating the distance-difficulty hypothesis. It acknowledges that attentiveness can vary at the screen-by-respondent level and accommodates individuals with different traits who may exhibit distinct levels of attentiveness. Simultaneously addresses a wide range of response patterns that emerge due to careless responding^17^.

Speaking of interpretability, Effrosynidis and Arampatzis^18^ compared 12 variable selection methods and showed that ensemble-based method Reciprocal Ranking is the most effective whilst the best individual method appeared to be SHapley Additive exPlanations (SHAP). SHAP is a method used to provide explanations for the decision-making process of models, specifically focusing on understanding sample-level decisions^19^. Interpretability of the decisions made by the prediction model plays a major role in understanding machine learning (ML) algorithms and can be presented in various ways. To date, well-known approaches such as global interpretability and local interpretability exist and have been applied in several studies^20–24^, as well as alternative techniques like model-specific and model-agnostic approaches^25^. While global interpretability focuses on decisions made at a population level, local interpretability takes it even further by emphasizing decisions occurring on an individual level^26^. Since this article focuses on eliminating careless respondents where we focus on the characteristics of an individual, the latter approach is also applicable to our study.

In terms of the interpretability of the method, Liu et al.^27^ proposed a framework to predict and interpret patient satisfaction with Random Forest and local explanation method. However, we have not encountered any studies that include local interpretability of the methods dealing with the detection of careless respondents.

Schroeders et al.^2^ introduced a novel gradient boosting machine (GBM) model response time-based approach to identify careless respondents. This study compared the proposed model against traditional methods for identifying careless respondents.

The purpose of this paper was to examine prediction model interpretability techniques to evaluate decisions made by the proposed model at the single participant level and to identify factors that mislead the model, which consequently results in misclassifications.

## Materials and methods

The data used in our study is publicly accessible through the Open Science Framework (OSF) repository^28^ , the link is provided in the “Data availability” section. It was collected by Schroeders et al.^2^ as part of their study on the detection of careless respondents. More specifically, they conducted a 3-month web-based survey study in the first third of 2020. Participants were randomly assigned into two groups. According to the study Schroeders et al.^2^, in the first group, participants answered given questions after carefully considering all given options (regular respondents), the participants of the second group were required to respond to the same questions in a speedy, careless manner (careless respondents). Therefore, the type of careless responding in this study refers to participants who were instructed to respond to survey questions in a speedy and careless manner (For more see Schroeders et al.^2^).

Simultaneously, authors were collecting demographic data (such as age, gender, profession and similar) and data that examined participants’ personality traits such as honesty-humility, emotionality, extraversion, agreeableness, conscientiousness and openness to experience. Authors additionally tracked and stored response times on each section of the questionnaire (six personality trait sections of 10 items).

In this study, we examined the option of using heterogeneous data to explain the characteristics of careless respondents. For that purpose, we compared the performance of the prediction models built under the following scenarios:

- Raw data as the responses (some of them reversed) to the questionnaire questions (*resp*, 60 variables)
- Data that provide information about the time of answering each section of questions (*rt*, six variables)
- Only indices for careless responding (*Careless*, 13 variables)
- Demographic data (*dem*, four variables)
- Combination pairs of resp, rt and dem (*resp_dem*, *rt_dem*, *resp_rt*)
- Data consisting of all three sources of data in a single dataset (*all*)
- Extracted data representing different indices of careless responding (data calculated using data-driven detection mechanisms to detect careless respondents), including data consisting of all three types of data (*all_extracted*)

In the study by Schroeders et al.^2^, the highest average balanced accuracy was achieved by the GBM prediction model built on a combination pair *resp_rt* (0.66 ± 0.06). The same combination pair *resp_rt* was used in model training and represented a baseline model of the first part of this study. In comparison with the original study by Schroeders et al., we also used extracted indices of careless responding (i.e. variables calculated from the available raw data by using the established indices of careless responding to detect careless respondents). It needs to be noted that the original study mentions the possibility of using indices of careless responding to build prediction models, but they also report that prediction performance gains were insignificant in the case of using additional indices of careless responding.

However, in this study, we use prediction model interpretability approaches to analyze to what extent the indices of careless responding can help us understand what the characteristics of the careless respondents are as well as why some predictions of the prediction model are wrong.

### Experimental setup

Initially, the source code for data cleaning was obtained from the repository proposed in the study conducted by Schroeders et al.^2^. Additionally, we added lines of code to create a subset of demographic data, a subset that is comprised of a combination of response time and demographic data and a subset with responses and demographic data. Similarly, a dataset with all variables and additional indices of careless responding was created.

According to Schroeders et al., the online survey was conducted using the SoSci-Survey tool from February 2020 to April 2020^2^. Schroeders et al. reported that a total of 605 respondents took part in the test, with 361 participants under normal conditions and 244 participants under conditions where they were not paying careful attention. Among the respondents, approximately two-thirds were female, while around one-third were male, and a small percentage identified as diverse. The average age of the participants was 43.1 years (+ 17.8). The composition of the sample, combining both regular and careless conditions, consisted of 28.1% students, 2.8% manual workers, 39.3% employees, 5% self-employed individuals, 16.5% retired individuals, and 8.3% belonging to other categories^2^.

The GBM model was evaluated using tenfold cross-validation repeated a hundred times, each iteration was performed with a different seed number, which assures the reproducibility of results. Training (n = 420) and test (n = 185) set were built by sampling without replacement each time with a different seed number. During the validation process, the following hyper-parameters were evaluated: interaction depth, the minimum number of observations in trees at the leaf level, the number of trees and shrinkage learning rate. Performance was displayed as a balanced accuracy. For the purpose of the case study, the GBM model was trained on a subset consisting of all data and indices of careless responding (all_extracted) with the seed number set to one. Model fit was stored, as well as training and test set. Dalex explainer was trained on the training set and later utilized within a supplementary web application.

### Evaluation metrics

The following evaluation metrics were included in this study: balanced accuracy for comparing prediction models and others such as the area under the curve (AUC), F1-score, sensitivity, and specificity that are included in the web application. Majority of them can be calculated through a confussion matrix (Table 1).

**Table 1:** Confussion matrix.

		Actual	
		Positive	Negative
Predicted	Positive	True positive (TP)	False negative (FN)
	Negative	False positive (FP)	True negative (TN)
$$Precision = \frac{TP}{TP+FP}$$			

Sensitivity is a metric that measures the proportion of actual positive samples (true positive—TP) that are correctly predicted by the prediction model.

$$\begin{aligned} Sensitivity=\frac{TP}{TP+FN}. \end{aligned}$$

On the contrary, specificity is a metric that measures the proportion of actual negative samples (true negative—TN) that are correctly predicted.

$$\begin{aligned} Specificity=\frac{TN}{TN+FP}. \end{aligned}$$

Balanced accuracy is the metric that averages sensitivity and specificity.

$$\begin{aligned} \text {Balanced accuracy}=\frac{Sensitivity+Specificity}{2} \end{aligned}$$

F1-score is a metric based on precision and sensitivity.

$$\begin{aligned} F1=\frac{2*Precision}{Precision+Sensitivity}. \end{aligned}$$

The area under the curve is calculated as the area below the Receiver Operating Characteristic (ROC) curve. The ROC curve is displayed on a two-dimensional graph, where data points are determined by sensitivity (y-axis) and (1 − specificity) (x-axis) for all possible cutoff values^29^.

### Gradient boosting machine

Gradient boosting machine (GBM) is an ensemble machine learning method that combines the knowledge of several weak prediction models. The ensemble of models is built sequentially, where each subsequent model corrects the mistakes made by previous models, therefore, minimizes a loss function^30,31^.

### Indices of careless responding

Package that deals with indices of careless responding, known as Careless package^32^ for statistical language R, was used to calculate the indices of careless responding needed for generating *all_extracted* dataset. This library provides different data-driven detection mechanisms to detect careless respondents (noted as indices of careless responding in this paper): psychometric synonym (res_psycsyn), psychometric antonym (res_psychant), longstring (str), average longstring (avgstr), intra-individual response variability (irv), Mahalanobis distance (res_mahad) and Even-Odd consistency index (res_evenodd).

The following indices of careless responding were used as additional variables in the dataset: *psychometric synonym (psychsyn)* score is a measure of strongly positively correlated item pairs. Each respondent’s score is calculated as the within-person correlation between corresponding item pairs. In contrast, *psychometric antonym (psychant)* score is a measure of strongly negatively correlated item pairs, whereas the score at the respondent’s level is therefore determined in an identical way^1^. *Longstring*, also known as longstring index, represents several consecutive identical responses made by a respondent. Its variation, average longstring, represents an average number of consecutive identical responses for each respondent^4^. Another metric, *intra-individual response variability* (*irv*) is a measured standard deviation of consecutive respondent’s responses across all item responses. It is considered as an extension of longstring index^10^. *Mahalanobis distance* (*res_mahad*) measures “multivariate distance between respondent’s response vector and the vector of sample means”^1^. *Even-Odd consistency index* (*res_evenodd*) is a measure that yields within-person product-moment correlation between even numbered and odd numbered half scale scores among all scores^4^.

### Shapley additive explanations

In this subsection, we describe a prediction model interpretability technique that was used to obtain an explanation of the model predictions.

Shapley additive explanations, also known as SHAP, is a technique to explain models’ decisions on an individual sample level. It applies principles of game theory to compute Shapley values which measure the variables’ influence on the model’s prediction. Shapley value is the result of averaging the variable’s marginal contributions to every possible sample prediction and is determined with the following formulation^19,33^:

$$\begin{aligned} \phi _i=\sum _{S\subseteq F\backslash \{i\}}^{} \frac{|S|!(|F|-|S|-1)!}{|F|!}[f_{S\cup \{i\}}(x_{S\cup \{i\}})-f_S(X_S)], \end{aligned}$$

where F is the set of all variables, $f_{S\cup \{i\}}$ is the model trained with the variable $i$ included, $f_S$ is the model with the variable withheld and $x_S$ are the values of the variables within the subset $S$. For a detailed explanation, please check the publication of Lundberg et al.^34^. A higher positive shapley value suggests that a variable has greater importance in determining a positive outcome, while a higher negative shapley value indicates greater importance in determining a negative outcome. When a shapley value is equal to zero, it means that this variable does not contribute anything or is not directly connected to the decision^35^.

### Supplementary web application

Additionally, we built a Shiny application^36^ that allows comparison of the proposed GBM model, built on data consisting of responses, demographic information, and Indices of careless responding. In a separate tab of the web application, we offer an insight into the local interpretability of the GBM model built on all_extracted data, including response times, as well as other indices of careless responding.

In a comparison, GBM performance is compared against the following indices of careless responding: *psychometric synonym score (psychsyn)*, *psychometric antonym score (psychant)*, *longstring* and *longstring (Average)*. Optionally, we added several decision thresholds that present boundaries between predicted careless respondents and regular respondents. These thresholds can be interactively modified for each careless metric to obtain the highest possible performance. Performance can be compared with evaluation metrics such as an area under a curve (auc), F1-score (F1), sensitivity (sens) and specificity (spec).

Moreover, we provided additional explanations of decisions that were made by the GBM model. Web application (second tab) offers a visualization of the SHAP (Shapely) values which provide an overview of local interpretability^34^. Local interpretability can be displayed per each response on demand. We included a filtering option that narrows response selection of responses that were either correctly predicted careless responses (true positives—TP), incorrectly predicted careless responses (false positives—FP), correctly predicted regular responses (true negatives—TN) or incorrectly predicted regular responses (false negatives—FN).

The web application is available at: https://lkopitar.shinyapps.io/CarelessGBMShap/.

### Statistical analysis

All experiments were conducted in the programming language R^37^, using three main packages: *gbm*^30,38^, *Careless*^39^, *Shiny*^40,41^ and *DALEX*^42^. The classification performance of the prediction models was measured as the area under the ROC curve with corresponding standard deviations.

Quartile representation was used in the figures produced by the Dalex package (see Supplementary Figs. S1, S2) and in the figure displaying a comparison of model performance between models built on different sets of data. Dalex uses boxplots by default, offering no options to change the representation to any other type. The figure of comparison of models’ performances contains performance points, consequently, we found boxplot to be the most suitable method for presenting such information. All remaining figures are based on average contributions with provided confidence intervals (95% CI) with an emphasis on the demonstration of the variable importance.

The source code for all experiments and supplementary web application is documented and publicly available at the following URL: https://github.com/lkopitar/Careless_SHAP.

### Ethics declarations

Informed Consent not needed, since the data used in this study is publicly available and previously published.

## Results

**Figure 1:**
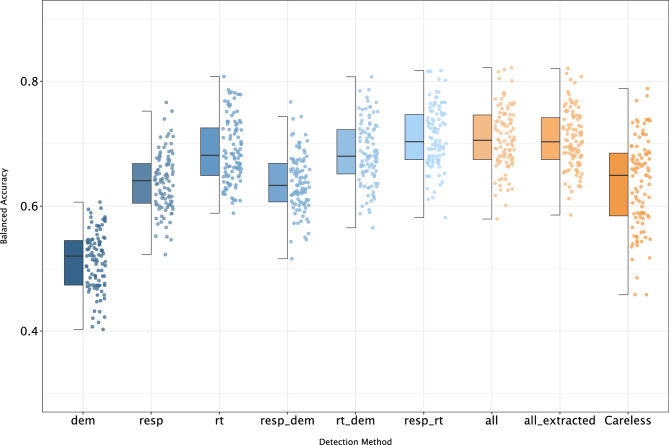
Comparison of models’ performances (balanced accuracy) of different detection methods (dem-demographic; resp-response; rt-response time; and other as described in “Materials and methods”).

Based on balanced accuracy, the GBM model built solely on response time data (0.637 ± 0.044) performed slightly better than the model built on responses alone (0.604 ± 0.042) (Fig. 1). Demographic data did not provide enough useful information for adequate prediction performance (0.518 ± 0.046) , as well as the subset of containing only indices for careless responding (0.589 ± 0.061). Furthermore, fusing demographic and response data brings no significant difference in model performance (0.601 ± 0.038) compared with the performance reached by response-only data. Similar observations can be noticed when the GBM model is built on a combination of response time and demographic data (0.631 ± 0.042) compared with response time data alone (0.637 ± 0.044). While previous pair combination models, with the addition of demographic data, did not contribute to overall performance, merging response and response time data (baseline model) apparently caused a slightly bigger, yet still insignificant shift toward higher performance (0.645 ± 0.045). Prediction models built on all types of data, including or excluding extracted information, did not result in any additional significant increase in performance (Fig. 1).

Since the performance of a baseline GBM model could not be significantly improved, we questioned whether indices of careless responding can provide any useful information and reveal any hidden patterns in decisions made by the baseline model. Due to that reason, we decided to examine the explainability of the prediction model built on *all_extracted*, which will reveal the characteristics of careless respondents in survey data.

More information on the interpretability of the careless respondents’ prediction model used in our case study can be found in the Supplementary material.

### Comparison of SHAP contributions

Comparing SHAP contributions under different circumstances can reveal the contribution of variables in distinguishing careless respondents from regular respondents. Furthermore, it indicates a path for reducing the cost that false negatives provide. Three comparisons were conducted: comparison of contributions of correctly predicted careless respondents and correctly predicted regular respondents, comparison of contributions of correctly and incorrectly predicted careless respondents, and finally, comparison of Shapley values of models built on data with and without indices of careless responding.

#### Comparison 1: Correct decisions of GBM model among careless and regular respondents

Opposite average contribution signifies a positive contribution of the particular variable for regular respondents and the same time negative contribution of the particular for careless respondents, or the other way around. Variables with the opposite average contribution among correctly predicted careless respondents and correctly predicted regular respondents were psychometric synonym (res_psycsyn), “I would never take a bribe, even if it was a lot” (HE01_36), time spent on section 5 (time_p5) and other such as the total intra-individual response variability (irvTotal), average length of consecutive identical responses (avgstr), “I feel strong emotions when someone close to me leaves for an extended period of time.” (”HE01_47 ), time spent on section 4 (time_p4), “I prefer to do whatever comes to my mind than stick to a plan”. (reversed) (HE01_56_r), time spent on section 3 (time_p3). The largest absolute difference in average contributions was shown in res_psycsyn ($\Delta _{contribution}=0.112$), time_p5 ($\Delta _{contribution}=0.081$), HE01_36 ($\Delta _{contribution}=0.054$) and time_p3 ($\Delta _{contribution}=0.030$).

**Figure 2:**
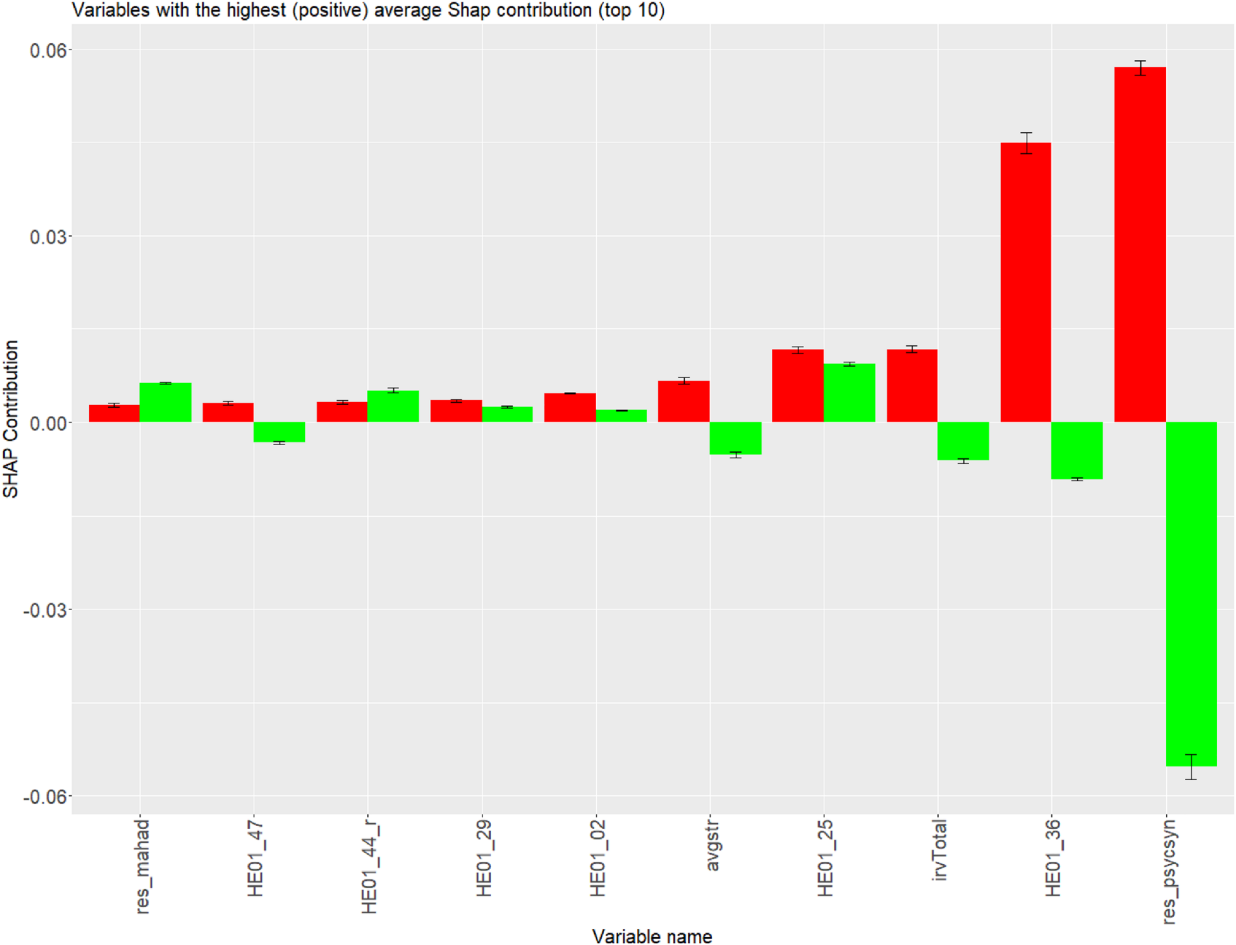
Comparison of variables’ contributions among correctly predicted careless respondents (in red) and regular respondents (in green). Variables are ordered by average contributions of correctly predicted careless respondents (Ascending order). Only the top 10 are displayed.

**Figure 3:**
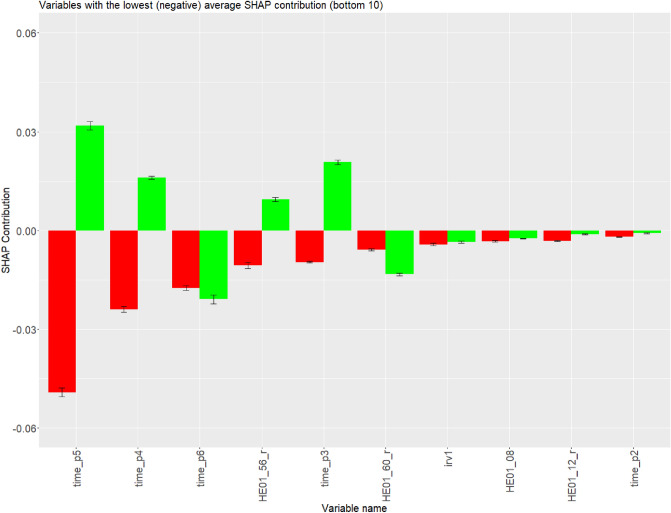
Comparison of variables’ contributions among correctly predicted careless respondents (in red) and regular respondents (in green). Variables are ordered by average contributions of correctly predicted careless respondents (Descending order). Only the bottom 10 are displayed.

On the contrary, although variables such as time spent on section 6 (*time_p6*), “If I had the opportunity, I would love to attend a classical music concert.” (*HE01_25*) and “I would be tempted to use counterfeit money if I could be sure of getting away with it. (reversed)” (*HE01_60_r*) have some impact on prediction performance, including significant average difference between correctly predicted careless respondents and correctly predicted regular respondents (Figs. 2, 3), but unfortunately the same, either positive or negative contribution simultaneously. Figure 2 demonstrates comparison of variables’ positive contributions among correctly predicted careless respondents and regular respondents, whereas Fig. 3 demonstrates comparison of variables’ negative contributions among correctly predicted careless respondents and regular respondents.

#### Comparison 2: Correct/incorrect decisions of GBM model in careless respondents

**Figure 4:**
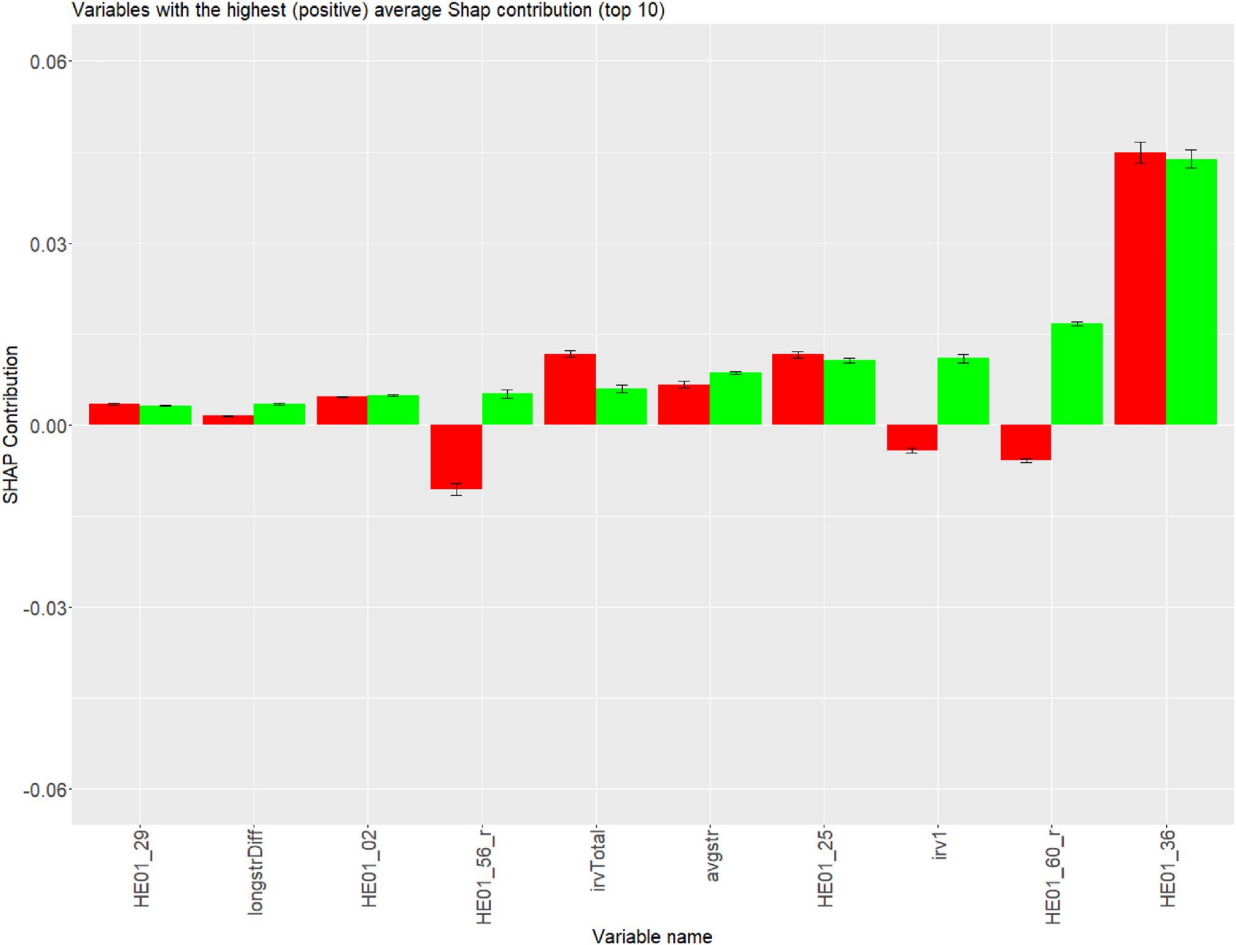
Comparison of correctly (in red) and incorrectly (in green) predicted careless respondents. Variables are ordered by average contributions of incorrectly predicted careless respondents (Ascending order). Only the top 10 are displayed.

**Figure 5:**
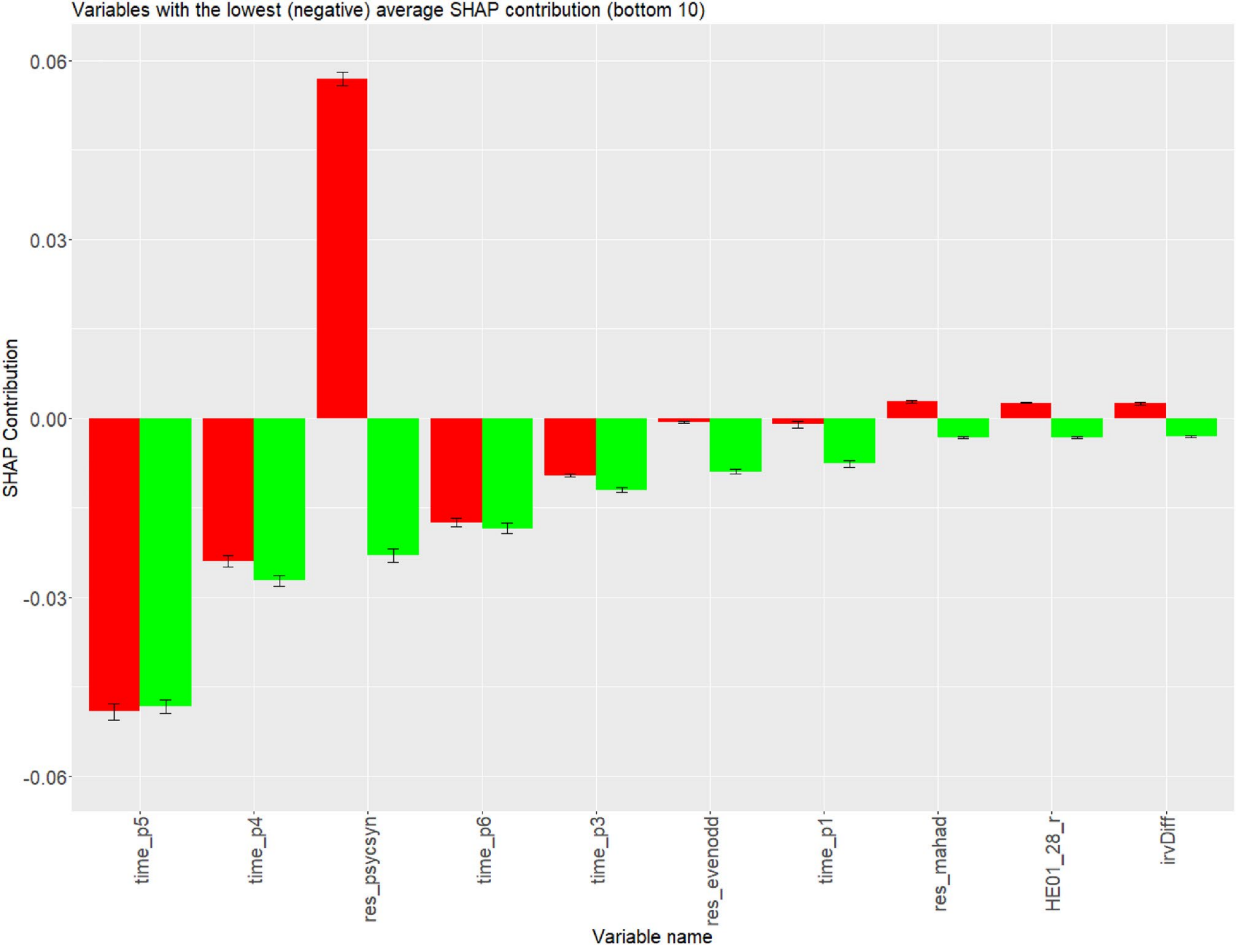
Comparison of correctly (in red) and incorrectly (in green) predicted careless respondents. Variables are ordered by average contributions of incorrectly predicted careless respondents (descending order). Only the bottom 10 are displayed.

Five the most influential variables were “I would never take a bribe, even if it was a lot” (*HE01_36*) (Fig. 4), *time_p5*, *res_psycsyn*, *time_p4* and *time_p6* (Fig. 5). Among these, only *res_psycsyn* and *time_p4* resulted in a significant average difference in contribution to the final prediction. Variable *time_p4* was related to negative contribution in the majority of cases, whereas *res_psycsyn* contributed positively in cases where careless respondents were correctly classified and contributed negatively in cases where careless respondents were misclassified (Fig. 5).

#### Comparison 3: Shapley values of the first respondent with and without indices of careless responding (all, all_extracted)

The aim of this experiment was to examine the explicability of the model where additional indices of careless responding are available to explain the predictions of the GBM model. The first respondent examined belonged to a group of regular respondents, and it was correctly predicted by the GBM model. In the use case example, we observed that it was a female respondent, 65 years old whose average response time was 65820.04 ms ($95\%$ CI 56700.90–74939.18). Throughout the entire dataset, the average response time spent on one of six sections for regular respondents was 57755.95 ms ($95\%$ CI 55776.48–59735.43), whereas for careless respondents it was approximately 14 s lower at 43271.06 ($95\%$ CI 40566.27–45975.84).

As mentioned earlier, we compared the prediction model interpretability results for this use case respondent before and after the addition of indices of careless responding. Among ten of the most influential variables, even the addition of indices of careless responding, did not replace *time_p5* as the most influential variable. The most influential variable from the set of indices of careless responding was *res_psycsyn* overtook the position of the second most influential variable, the statement “I would be tempted to use counterfeit money if I could be sure of getting away with it. (reversed)” (*HE01_60_r*) (raw answers to question number 60) dropped to the seventh place, just after *time_p1*, *time_p6*, *time_p4* and careless metric Mahalanobis distance (*res_mahad*). In addition, another marginal decrease was observed for a variable “If I had the opportunity, I would love to attend a classical music concert” (*HE01_25*) that dropped by two positions and was ranked as the eighth most important variable.

Generally speaking, considering the changes (before and after the addition of indices of careless responding) in average contributions of specific variables among all respondents, only *time_p6*, “I would be tempted to use counterfeit money if I could be sure of getting away with it. (reversed)” (*HE01_60_r*) and “If I had the opportunity, I would love to attend a classical music concert” (*HE01_25*) experienced a significant decrease in contribution, while *time_p3* was the only variable whose average contribution increased (Table 2). Indices of careless responding, such as *res_psycsyn* and *irv1* (not included in Table 2, since it is present only in *all_extracted*), contributed positively in favour of careless respondents, where time variables (*time_p1*, *time_p4*, *time_p5*, as well as *time_p6* and *time_p3*) leaned towards the decision of regular respondents.

**Table 2:** Comparison of average Shapley values with and without indices of careless responding. Average contributions are provided with 95% CI (in square brackets). Variable, where significant difference is observed, are marked in bold.

Variable	All	All_extracted
Time_p5	$-0.0569$ [$-0.0583$, $-0.0555$]	$-0.0539$ [$-0.0562$, $-0.0517$]
Time_p1	$-0.0222$ [$-0.0230$, $-0.0214$]	$-0.0236$ [$-0.0249$, $-0.0223$]
**Time_p6**	$-0.0246$ [$-0.0257$, $-0.0234$]	$-0.0194$ [$-0.0203$, $-0.0185$]
time_p4	$-0.0165$ [$-0.0173$, $-0.0156$]	$-0.0177$ [$-0.0185$, $-0.0169$]
**HE01_60_r**	0.0263 [0.0258, 0.0268]	0.0167 [0.0163, 0.0170]
**Time_p3**	$-0.0078$ [$-0.0081$, $-0.0075$]	$-0.0099$ [$-0.0102$, $-0.0096$]
**HE01_25**	0.0130 [0.0127, 0.0133]	0.0104 [0.0101, 0.0107]

### Summary of SHAP contribution comparison

The characteristics of careless respondents and the appropriateness of variables to be included in the prediction model will be assessed in the following paragraphs. The table of the appropriateness of variables for fitting a prediction model according to Shapley values is located below (Table 3). Variables were ranked with plus and minus signs, where plus sign signifies an appropriate variable and the minus signifies an inappropriate variable. Signs and the amount of signs are assigned depending on the topic of comparison, significance and level of contribution. The number of assigned signs is included within the parenthesis after the grading of each variable is explained and in Table 3. Figures of two comparisons (Comparison 1 and Comparison 2) display the top and bottom 10 variables according to the average contribution. Based on that, if a variable appears within five (top and bottom) of the most influential variables and the average difference among groups is not significant, then such a variable gets assigned three plus/minus signs. In case a variable, based on the average SHAP contribution, appears within the 5 most influential variables but the difference among groups is significant then such a variable gets assigned two signs, while in other scenarios only a single sign. The last criterion is based on the results of the average contribution of models with and without indices of careless responding (Table 2). For that criteria, a single plus sign is assigned to the variable that displays stability, and minus for a variable that characterizes instability after including indices of careless responding. Here we had chosen an inclusion criterion where the average contribution of either group should be at least 0.01, rounded on three decimal places precisely. The overall level of appropriateness is then determined by merging signs from all three comparisons together, where merging one plus and one minus sign displays the neutral level of variable appropriateness. The maximum amount of signs is limited to three. The overall level of appropriateness was not calculated for variables with only one rated criterion.

The study of Gramegna and Giudici^43^ has shown that the use of Shapley values can provide more accurate information on variable selection. In our study, the average SHAP contributions of variables, where correct decisions of the GBM model among careless respondents and regular respondents were observed (Comparison 1), showed that the more opposite contributions of a variable are between these two groups (regular respondents and careless respondents), the more appropriate the variable might be for fitting the GBM model when it comes to distinguishing between careless respondents and regular respondents. Attributes that fall into that category are: *res_psycsyn* (+++), “I would never take a bribe, even if it was a lot” (*HE01_36*) (+++), *time_p3* (+++), *time_p4* (+++), *time_p5* (+++), “I prefer to do whatever comes to my mind than stick to a plan (reversed)”. (*HE01_56_r*) (+++), *avgstr* (+++) and *irvTotal* (+++), and potentially “I feel strong emotions when someone close to me leaves for an extended period of time.” (*HE01_47*) (+). Variables, whose average contribution is within five most influential variables and shows significant difference between these two groups but simultaneously both contributions are either negative or positive, are “If I had the opportunity, I would love to attend a classical music concert”g variables are placed below five the most influential (top/bottom) variables: *res_mahad* (−), “I make a lot of mistakes because I don’t think before I act (reversed)” (”*HE01_44_r*) (−), “When it comes to physical dangers, I am very anxious.” (*HE01_29*) (−), “I plan ahead and organize so that there is no time pressure at the last minute.” (*HE01_02*) (−), “I would be tempted to use counterfeit money if I could be sure of getting away with it. (reversed)” (*HE01_60_r*) (−), *irv1* (−), “I often push myself very hard when I am trying to achieve a goal.” (*HE01_08*) (−), “If I knew I would never get caught, I would be willing to steal a million. (”*HE01_12_r*) (−) and *time_p2* (−).

Comparison of the average contributions of correct and incorrect decisions of the GBM model among careless respondents (Comparison 2) revealed attributes that GBM depends on, therefore, potentially distorts the robustness of the model. These variables can be recognized by comparing their average distributions among correctly predicted and incorrectly predicted careless respondents. The more opposite are contributions, the higher the chance that we might deal with an inappropriate variable. Attributes that potentially fall into that category are irv1 ($- - -$), “I would be tempted to use counterfeit money if I could be sure of getting away with it. (reversed)” (*HE01_60_r*) ($- - -$) (Fig. 4) and *res_psycsyn* ($- - -$) (Fig. 5), potentially even *res_mahad* (−), “I am of the opinion that I am not popular. (reversed)” (*HE01_28_r*) (−), “I prefer to do whatever comes to my mind than stick to a plan (reversed)”. (*HE01_56_r*) (−), and *irvDiff* (−). It is even more probable that *HE01_60_r* and especially *irv1* are the main candidates for an inappropriate variable due to weaker (negatively oriented) average contribution displayed in comparison dealt with correctly predicted careless respondents and regular respondents (see Comparison 1). Variable “I would never take a bribe, even if it was a lot” (*HE01_36*) (+++), as well as “If I had the opportunity, I would love to attend a classical music concert” (*HE01_25*) (+++), *time_p5* (+++), *time_p6* (+++), *avgstr* (++), *time_p4* (++) and *time_p3* (++), less likely “When it comes to physical dangers, I am very anxious.” (*HE01_29*) (+), *longDiff* (+), *HE01_02* (+), *irvTotal* (+), *res_evenodd* (+), *time_p1* (+) displayed stable (average) contribution over both groups, correctly and incorrectly classified careless respondents (see Comparison 2). This leads a step closer to the confirmation of the appropriateness of the variable for fitting a model for detecting careless respondents. The explanation is that the opposite average contribution of the specific variable might present a cause for Type II Error. While *res_psycsyn* and *HE01_56_r* might be suitable for separating careless respondents and regular respondents (according to Comparison 1; see Figs. 2 and 3), it is an option that both variables increase the chances that actual careless respondents are classified as regular respondents (Comparison 2; see Figs. 4 and 5), similar logic applies also to *HE01_25*.

**Table 3:** Appropriateness of variables for fitting a prediction model according to Shapley values. Signs that are used are following: (−) innapropriate, ($+$) appropriate, (0) unsure, where levels can be: −−−, −−, −, 0, $+$, $++$ and $+++$. More signs indicate a higher chance for a variable to be appropriate/inappropriate.

Variable	Meaning	Comparison 1	Comparison 2	Criteria 3	Level of variable appropriateness
res_psychsyn	“I would be tempted to use counterfeit money if I could be sure of getting away with it. (reversed)”	$+++$	−−−		0
res_mahad	Mahalanobis distance	−	−		−−
res_evenodd	Even-Odd consistency index		$+$		*X*
HE01_02	I plan ahead and organize so that there is no time pressure at the last minute.	−	$+$		0
HE01_08	I often push myself very hard when I am trying to achieve a goal.	−			*X*
HE01_25	If I had the opportunity, I would love to attend a classical music concert.	−−	$+++$	−	0
HE01_29	When it comes to physical dangers, I am very anxious.	−	$+$		0
HE01_36	I would never take a bribe, even if it was a lot.	$+++$	$+++$		$+++$
HE01_47	I feel strong emotions when someone close to me leaves for an extended period of time.	$+$			*X*
HE01_12_r	If I knew I would never get caught, I would be willing to steal a million (reversed)	−			*X*
HE01_28_r	I am of the opinion that I am not popular. (reversed)		−		*X*
HE01_44_r	I make a lot of mistakes because I don’t think before I act (reversed)	−			*X*
HE01_56_r	I prefer to do whatever comes to my mind than stick to a plan (reversed)	$+++$	−		$++$
HE01_60_r	I would be tempted to use counterfeit money if I could be sure of getting away with it (reversed).	−	−−−	−	−−−
time_p1	Time spent on section 1		$+$	$+$	$++$
time_p2	Time spent on section 2	−			*X*
time_p3	Time spent on section 3	$+++$	$++$	−	$+++$
time_p4	Time spent on section 4	$+++$	$++$	$+$	$+++$
time_p5	Time spent on section 5	$+++$	$+++$	$+$	$+++$
time_p6	Time spent on section 6	−−−	$+++$		0
avgstr	Average longstring	$+++$	$++$		$+++$
irvTotal	Total intra-individual response variability	$+++$	$+$		$+++$
irv1	Intra-individual response variability of the first section	−	−−−		−−−
longDiff	Difference in longstrings between the first and the last section		$+$		*X*
irvDiff	Difference in intra-individual response variabilities betweenthe first and the last section		−		*X*

Further, the impact of indices of careless responding was evaluated. On average, the addition of indices of careless responding, specifically *res_psycsyn*, *res_mahad* and *irv1*, did not cause any obvious inverted changes in contributions of any existent most influential attributes (Table 2). On top of that, half of the time-related variables (time_p1 (+), time_p4 (+), time_p5 (+)) remained stable even afterward (insignificant difference in contributions), thus, if we relate to the Comparison 2 (Fig. 5), we can confirm the appropriateness of *time_p5*, *time_p4* and potentially even *time_p1*.

## Discussion

A prediction model based on response time performed with a higher average accuracy in comparison to models built on demographic data or responses alone. However, significant differences among them were not observed. While we expected that data fusion will boost prediction performance, evaluating models built on fused data did not bring any significant improvements.

Our study incorporates results of various approaches, including the utilization of indices of careless responding, as a variable. The distinctions and comparisons among these approaches are detailed in Schroeders et al.^2^.

Variables such as the question “would never take a bribe, even if it was a lot” (*HE01_36*), “I prefer to do whatever comes to my mind than stick to a plan.” (*HE01_56*), majority of time-related variables, indices of careless responding such as average longstring (avgstr), total intra-individual response variability (irvTotal) were ranked as a medium to highly appropriate for inclusion. The reason for *HE01_36* and *HE01_56* to be ranked among these variables might be due to the content of the questions, which requires regular respondents to read and understand it at first. Time-related variables include response times, where it is expected that careless respondents proceeds faster through questions than regular respondents, even though, on times it can fail to detect since participant might lose concentration, get distracted, stops for a moment during the survey, or pauses the survey^44^.

On the other hand, variables that might not bring much value to the decisions of the GBM model are “I would be tempted to use counterfeit money if I could be sure of getting away with it (reversed).” (*HE01_60_r*), *irv1*, as the section of irvTotal should be avoided, and considered intra-individual response variability instead and partially also Mahalanobis distance (*res_mahad*). While *HE01_36* and *HE01_56* present uncertainty behind the decision, *HE01_60_r* presents assurance and certainty of success, without the possibility of encountering additional problems. The result of ranking *res_mahad* with a medium level of inappropriateness is surprising since it is normally used for outlier detection^44^, but apparently, other variables contribute to the GBM model in adequate quantity that the contribution of *res_mahad* becomes insufficient.

In the case of the first respondent, as described in Comparison 3, the addition of indices of careless responding, *res_psycsyn* immediately became considered as the second most contributive variable. In that given example, its value was negative and close to none (*res_psycsyn=-0.1554*), which indicates a low within-person correlation between the identified item pairs. Accordingly, this index value suggests it goes for regular respondents, but SHAP recognized it as a variable that increases the chance of being a careless respondent (due to positive contribution). While the same variable is considered as one of the most influential variables as observed from the comparison of average contributions among correctly predicted careless respondents and regular respondents (Comparison 1; Fig. 2), GBM model should more emphasize other variables instead.

To confirm our speculations, for future work we might utilize frameworks such as Multiperturbation Shapley Analysis, which relies on game theory to estimate usefulness^45^, include Shapley values in a state-of-the-art integrated approaches for variable selection^43^, or implement more complex approaches, such as ensemble method Reciprocal Ranking^18^, adaptive variable selection approach ShapHT+^46^ and other.

Our technique was only used to analyze data from one database, which is one of the study’s limitations. Consequently, the generalizability of the results to broader populations or different datasets may be limited. In addition, our analysis relied only on a single prediction model, GBM. It would be interesting to apply our method using alternative models, such as logistic regression or random forest, which utilize bagging ensemble learning techniques.

## Conclussion

Completed questionnaires, prior to further analysis, should also undergo the process of eliminating careless respondents. In comparison to the referring study of Schroeders et. al., data fusion did not bring any significant improvements in the performance of the GBM model. The use case in this study further demonstrated that even though the psychometric synonym score demonstrates an immediate impact and is built with the intention of discovering careless respondents, in combination with other variables is not always the most ideal choice to be fit into a GBM model. Moreover, a variable that stores the answers to the question “I would never take a bribe, even if it was a lot” (*HE01_36*), average longstring (*avgStr*), total intra-individual response variability (*irvTotal*) as well as most response times (*time_p3*, *time_p4*, *time_p5*) are appropriate for detecting careless respondents. On the contrary, intra-individual response variability (*irv1*), the question that asks for an answer to “I would be tempted to use counterfeit money if I could be sure of getting away with it (Reversed).” (*HE01_60_r*) are better to be avoided.

The main contribution of this paper is in the interpretation of the decisions from the prediction model using Shapley values. We also showed that although additional variables do not bring better classification performance, can contribute to much more interpretable prediction models.

### Supplementary Information

Supplementary Information.

## Data Availability

The data, provided by Schroeders et al.^2^, used in our study is publicly accessible through the Open Science Framework (OSF) repository at https://osf.io/mct37/.

## References

[1] Meade AW, Craig SB (2012). Identifying careless responses in survey data. Psychol. Methods.

[2] Schroeders U, Schmidt C, Gnambs T (2021). Detecting careless responding in survey data using stochastic gradient boosting. Educ. Psychol. Meas..

[3] Credé M (2010). Random responding as a threat to the validity of effect size estimates in correlational research. Educ. Psychol. Meas..

[4] Johnson JA (2005). Ascertaining the validity of individual protocols from Web-based personality inventories. J. Res. Pers..

[5] Maniaci MR, Rogge RD (2014). Caring about carelessness: Participant inattention and its effects on research. J. Res. Pers..

[6] Huang JL, Curran PG, Keeney J, Poposki EM, DeShon RP (2012). Detecting and deterring insufficient effort responding to surveys. J. Bus. Psychol..

[7] Niessen ASM, Meijer RR, Tendeiro JN (2016). Detecting careless respondents in web-based questionnaires: Which method to use?. J. Res. Pers..

[8] Costa PT, McCrae RR (2008). The revised neo personality inventory (NEO-PI-R). SAGE Handb. Pers. Theory Assess..

[9] Ehlers, C., Greene-Shortridge, T., Weekley, J. & Zajack, M. The exploration of statistical methods in detecting random responding In *Annual meeting of the Society for Industrial/Organizational Psychology, Atlanta, GA* (2009).

[10] Dunn AM, Heggestad ED, Shanock LR, Theilgard N (2018). Intra-individual response variability as an indicator of insufficient effort responding: Comparison to other indicators and relationships with individual differences. J. Bus. Psychol..

[11] Dogan V (2018). A novel method for detecting careless respondents in survey data: Floodlight detection of careless respondents. J. Market. Anal..

[12] Marjanovic Z, Holden R, Struthers W, Cribbie R, Greenglass E (2015). The inter-item standard deviation (ISD): An index that discriminates between conscientious and random responders. Pers. Individ. Differ..

[13] Goldammer P, Annen H, Stöckli PL, Jonas K (2020). Careless responding in questionnaire measures: Detection, impact, and remedies. Leadersh. Q..

[14] Wind, S. & Wang, Y. Using mokken scaling techniques to explore carelessness in survey research. *Behav. Res. Methods* 1–46 (2022). 10.3758/s13428-022-01960-y 36131197

[15] Arias VB, Garrido L, Jenaro C, Martínez-Molina A, Arias B (2020). A little garbage in, lots of garbage out: Assessing the impact of careless responding in personality survey data. Behav. Res. Methods.

[16] Ulitzsch E, Yildirim-Erbasli SN, Gorgun G, Bulut O (2022). An explanatory mixture IRT model for careless and insufficient effort responding in self-report measures. Br. J. Math. Stat. Psychol..

[17] Ulitzsch E, Pohl S, Khorramdel L, Kroehne U, von Davier M (2022). A response-time-based latent response mixture model for identifying and modeling careless and insufficient effort responding in survey data. Psychometrika.

[18] Effrosynidis D, Arampatzis A (2021). An evaluation of feature selection methods for environmental data. Eco. Inform..

[19] Molnar, C. *Interpretable Machine Learning* (Lulu. com, 2020).

[20] Bratko I (1997). Machine learning: Between accuracy and interpretability. Learn. Netw. Stat..

[21] Stiglic G, Mertik M, Podgorelec V, Kokol P (2006). Using visual interpretation of small ensembles in microarray analysis. Proc. IEEE Symp. Comput. Based Med. Syst..

[22] Martens D, Huysmans J, Setiono R, Vanthienen J, Baesens B (2008). Rule extraction from support vector machines: An overview of issues and application in credit scoring. Stud. Comput. Intell..

[23] Hall, P., Gill, N., Kurka, M., Phan, W. & Bartz, A. Machine learning interpretability with h2o driverless ai. http://docs.h2o.ai (2019).

[24] Kopitar, L., Cilar, L., Kocbek, P. & Stiglic, G. Local vs. global interpretability of machine learning models in type 2 diabetes mellitus screening. In *International Workshop on Knowledge Representation for Health Care* 108–119 (organizationSpringer, 2019).

[25] Hinton, G., Vinyals, O. & Dean, J. Distilling the Knowledge in a Neural Network. arXiv 10.48550/arxiv.1503.02531 (2015). eprint1503.02531.

[26] Stiglic G (2020). Interpretability of machine learning-based prediction models in healthcare. Wiley Interdiscip. Rev. Data Mining Knowl. Discov..

[27] Liu, N., Kumara, S. & Reich, E. Explainable data-driven modeling of patient satisfaction survey data. In *Proceedings—2017 IEEE International Conference on Big Data, Big Data 2017*, 2018-January, 3869–3876. 10.1109/BigData.2017.8258391 (2017).

[28] Foster ED, Deardorff A (2017). Open science framework (OSF). J. Med. Libr. Assoc..

[29] Hanley JA, McNeil BJ (1982). The meaning and use of the area under a receiver operating characteristic (ROC) curve. Radiology.

[30] Friedman JH (2002). Stochastic gradient boosting. Comput. Stat. Data Anal..

[31] Sagi O, Rokach L (2018). Ensemble learning: A survey. Wiley Interdiscip. Rev. Data Mining Knowl. Discov..

[32] Yentes, R. D. & Wilhelm, F. Careless: Procedures for computing indices of careless responding (2021). R package version 1.2.1.

[33] Leslie D (2019). Understanding artificial intelligence ethics and safety: A guide for the responsible design and implementation of AI systems in the public sector, the alan turing institute. Zenodo.

[34] Lundberg, S. M. & Lee, S.-I. A unified approach to interpreting model predictions. *Adv. Neural Inf. Process. Systems* **30** (2017).

[35] Ma, S. & Tourani, R. Predictive and causal implications of using shapley value for model interpretation. In *Proceedings of the 2020 KDD Workshop on Causal Discovery, vol. 127 of Proceedings of Machine Learning Research*, 23–38 (PMLR, 2020).

[36] Campbell, M., Shiny, R. Dashboards. Learn RStudio IDE 99–112.10.1007/978-1-4842-4511-8_9 (2019).

[37] Stiglic G, Watson R, Cilar L (2019). R you ready? Using the R programme for statistical analysis and graphics. Res. Nurs. Health.

[38] Biecek P (2001). Greedy function approximation: A gradient boosting machine. Ann. Stat..

[39] Yentes R, Wilhelm F (2018). careless: Procedures for computing indices of careless responding. R Package Version.

[40] Chang, W. Package ‘shiny’—Web Application Framework for R Version. R package version (2016).

[41] Chang, W. *et al.* shiny: Web Application Framework for R (2021). R package version 1.6.0.

[42] Biecek P (2018). Dalex: Explainers for complex predictive models in r. J. Mach. Learn. Res..

[43] Gramegna A, Giudici P (2022). Shapley feature selection. FinTech.

[44] Ward, M. & Meade, A. W. Dealing with careless responding in survey data: Prevention, identification, and recommended best practices. *Annu. Rev. Psychol.* **74** (2023). 10.1146/annurev-psych-040422-045007 35973734

[45] Cohen, S., Ruppin, E. & Dror, G. Feature selection based on the shapley value. Other words **1**, 98Eqr (2005).

[46] Yin D, Chen D, Tang Y, Dong H, Li X (2022). Adaptive feature selection with Shapley and hypothetical testing: Case study of EEG feature engineering. Inf. Sci..

