# Supplementary material for: Using heterogeneous sources of data and interpretability of prediction models to explain the characteristics of careless respondents in survey data
Source: Sci Rep. 2023 Aug 17;13:13417. doi: 10.1038/s41598-023-40209-2 (PMC10435557; doi:10.1038/s41598-023-40209-2)
Supplement: Supplementary file 1 — Supplementary Information. [file 41598_2023_40209_MOESM1_ESM.pdf]

Supplementary information:

Using heterogeneous sources of data and interpretability of prediction models to explain the characteristics of careless respondents in survey data

Leon Kopitar [1,2,*] Gregor Stiglic [1,2,3]
[1] Faculty of Health Sciences, University of Maribor, Maribor, Slovenia
[2] Faculty of Electrical Engineering and Computer Science, University of Maribor, Maribor, Slovenia
[3] Usher Institute, University of Edinburgh, Edinburgh, UK
*leon.kopitar1@um.si

Case study: interpretability of careless respondents prediction model

In this case study, we want to demonstrate and reveal the underlying causes of incorrect decisions made by the GBM model. Local interpretability displays the contribution of attributes (utilizing Shapley values) that led to the specific decision by the prediction model - in this case, a wrong decision. These attributes are then suspected as incorrectly utilized or inappropriate to be fit by a GBM model. Based on exposed attribute contributions, we might pre-eliminate incorrectly predicted careless respondents (false positives) and thus additionally enhance the performance of the proposed prediction model.

Here we present the results from a comparison between the first listed questionnaire, which GBM model incorrectly predicted as Careless respondent (Supplementary Figure S1) and the first listed questionnaire, which GBM correctly predicted as Careless respondent (Supplementary Figure S2). Comparison is based on the top ten variables with the highest contribution.

In general, response time variable (*time_p5*) highly contributed to the decisions made in both cases. In a case where GBM predicted the outcome correctly (*time_p5 = 12280*) it gave a positive contribution, whereas in a case where a participant took much more time (*time_p5 = 73600*), this same variable contributed negatively. Most time-related variables (4/5, all except time_p1) made a positive contribution to the prediction of correctly predicted Careless responses, and at the same time, these identical variables made a negative contribution to the prediction of incorrectly predicted Careless response. Positive contributions point to increased chances of Careless respondent (positive outcome). Variable (*res_psycsyn*) in both completed questionnaires leaned towards a decision that questionnaires are indeed Careless respondents. Contribution is alike, despite unexpected information that the psychometric synonym index in an incorrectly predicted Careless response was negative, which already slightly satisfies a property of a positive psychometric antonym. However,

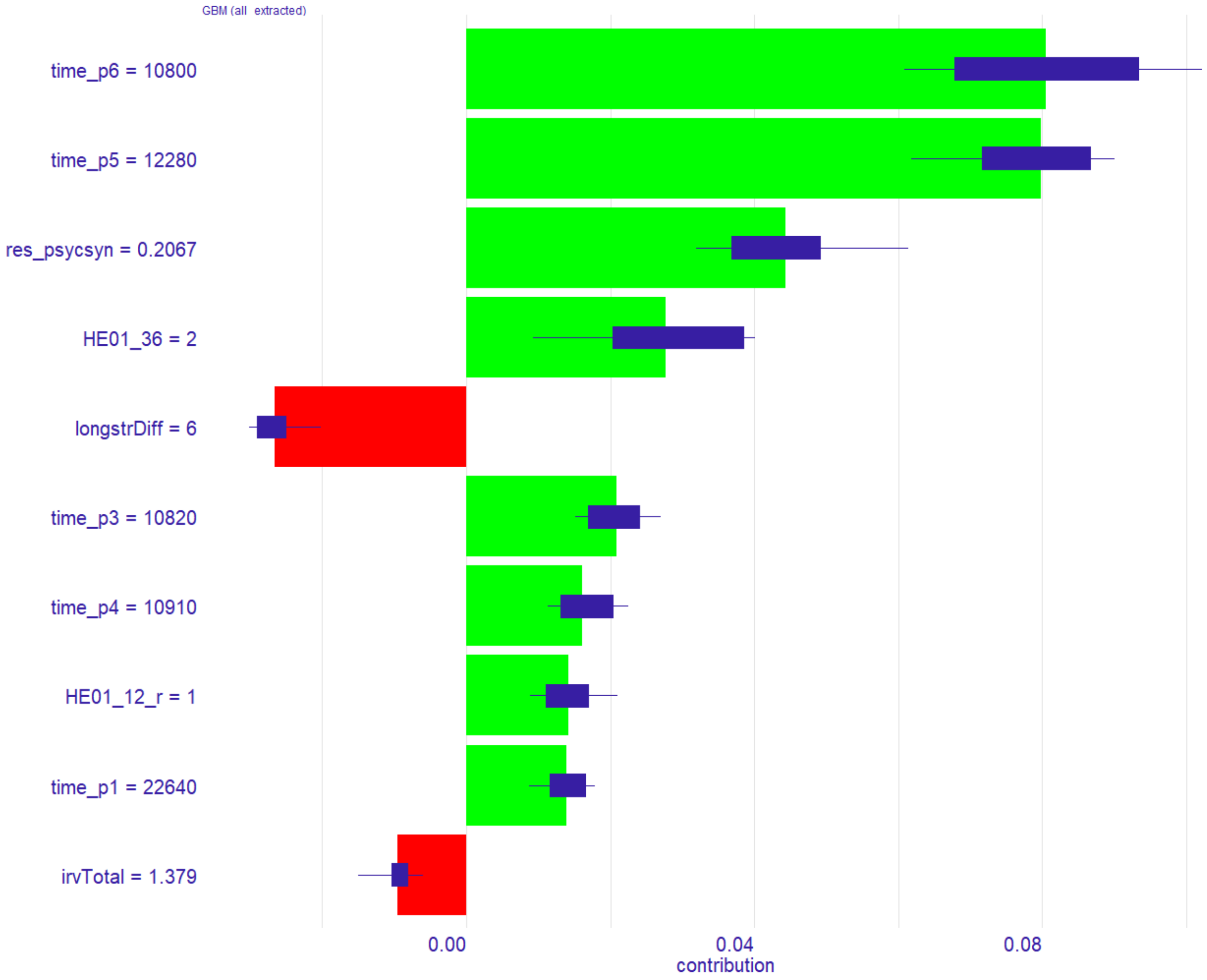

Supplementary Figure 1. First Careless respondent that was correctly classified by GBM model.

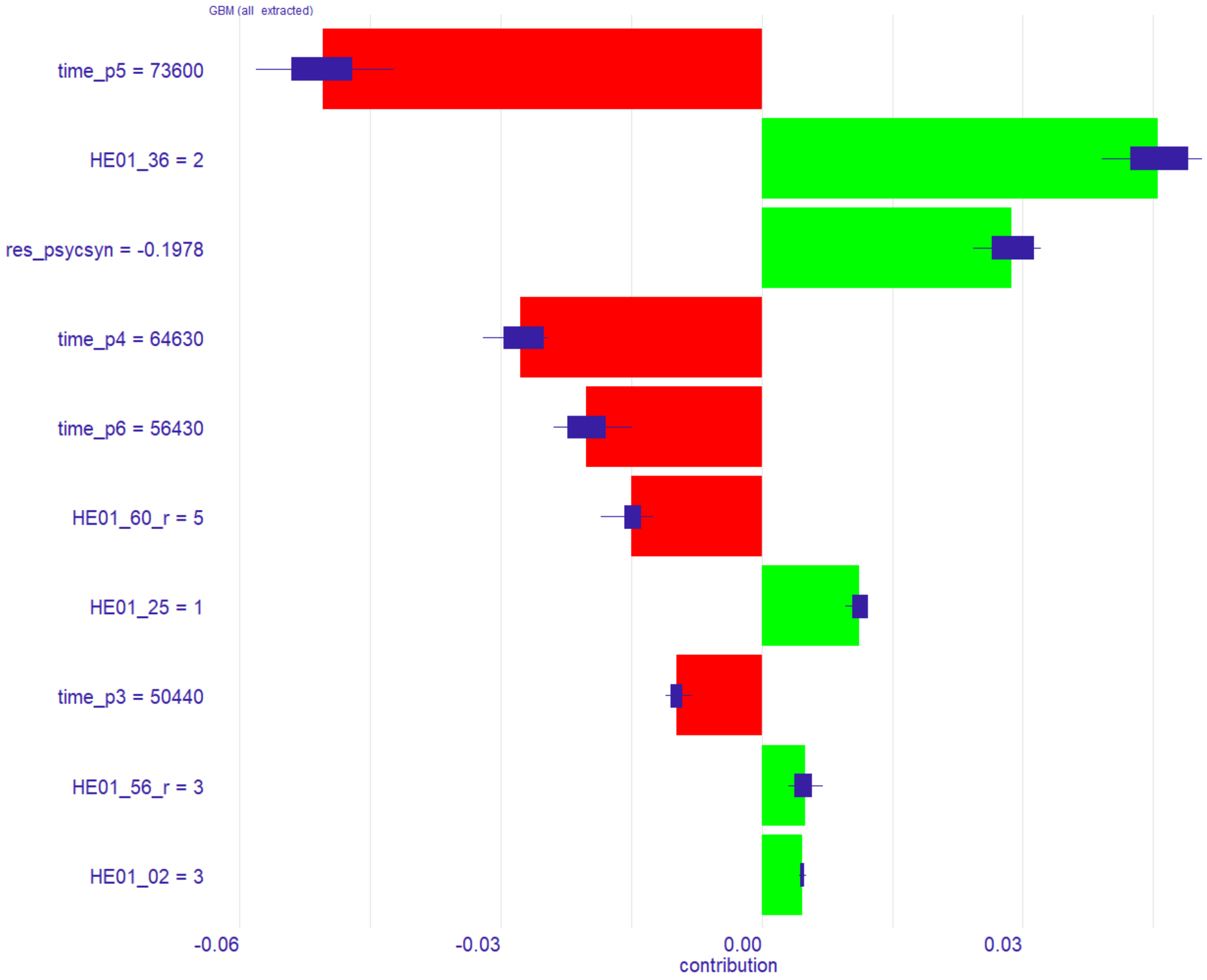

Supplementary Figure 2. First Careless respondent that was incorrectly classified by GBM model.
